# The Role of Glycosylation in Breast Cancer Metastasis and Cancer Control

**DOI:** 10.3389/fonc.2015.00219

**Published:** 2015-10-13

**Authors:** Alexandra C. Kölbl, Ulrich Andergassen, Udo Jeschke

**Affiliations:** ^1^Department of Obstetrics and Gynaecology, Ludwig-Maximilians-University of Munich, Munich, Germany

**Keywords:** glycosylation, breast cancer, prognosis, therapy, carbohydrates

## Abstract

Glycosylation and its correlation to the formation of remote metastasis in breast cancer had been an important scientific topic in the last 25 years. With the development of new analytical techniques, new insights were gained on the mechanisms underlying metastasis formation and the role of aberrant glycosylation within. Mucin-1 and Galectin were recognized as key players in glycosylation. Interestingly, aberrant carbohydrate structures seem to support the development of brain metastasis in breast cancer patients, as changes in glycosylation structures facilitate an overcoming of blood–brain barrier. Changes in the gene expression of glycosyltransferases are the leading cause for a modification of carbohydrate chains, so that also altered gene expression plays a role for glycosylation. In consequence, glycosylation and changes within can be useful for cancer diagnosis, determination of tumor stage, and prognosis, but can as well be targets for therapeutic strategies. Thus, further research on this topic would worthwhile for cancer combating.

## Glycosylation in Breast Cancer Metastasis – Past and Present

Almost 25 years ago, researchers found a correlation between altered glycosylation of primary breast cancer cells and the formation of lymph node metastasis. Back then, changes in glycosylation were measured by the binding of helix pomatia lectin (HPA) to cellular structures like alpha-*N*-acetylgalactosamine residues, for example, the Thomsen-nouvelle (Tn) epitope or blood group A antigen. A coherence between staining of paraffin embedded tissues with HPA and the development of metastasis, especially in lymph nodes could be shown, while no association could be proved with tumor size, histological grade, or patient age at time of diagnosis. HPA staining was regarded as a predictor for long-term prognosis (1, 2). Another scientific approach was the analysis of glycosylation via *Datura stramonium* agglutinin (DSA)-Sepharose column, in which was shown that metastasized carcinomas contain two times more DSA-binding oligosaccharides than the normal mammary gland, while primary carcinomas contain an intermediate amount (3). Only a few years later, the Tn and Thomsen–Friedenreich (T) epitopes were recognized as autoimmunogenic pancarcinoma antigens, playing a role in cell invasion and metastasis (4). In 2000, CA15-3, a serum marker for breast cancer, was also recognized to have functions in breast cancer metastasis (5). In the following years, more and more modifications of glycosyl structures became known, having to do with breast cancer metastasis, for example 2,6 sialylation, which also contributes to altered cell adhesion (6).

After summarizing former research results, in the following passage the most recent insights into glycosylation and its influence on breast cancer metastasis should be further illustrated.

In 2011, the correlation between circulating tumor cells (CTCs) and glycosylation was investigated. Bi-, Tri-, and Tetraantennary-sialyl-Lewis x epitopes were detected in blood samples of metastatic breast cancer patients and healthy persons by high-performance liquid chromatography and results were compared. Glycan levels were found to be higher in breast cancer patients with high CTC counts, than in samples in which only few CTCs were detected and in healthy controls, so that they could be regarded as a prognostic marker (7). Another important pathway in breast cancer metastasis is the glucosaminyl (*N*-acetyl) transferase 2 (GCNT2)-pathway. The GCNT2 gene is overexpressed in metastatic tumor samples, and an ectopic expression of this gene provokes enhanced cell detachment from the extracellular matrix (ECM), adhesion to endothelial cells, cell migration and invasion *in vitro*, and lung metastases in *in vivo* model systems. GCNT2 is also involved in epithelial to mesenchymal transition (EMT), changing the expression of E-cadherin at the protein level and it is an effector in the TGF-beta signaling cascade. Therefore, in the future it might also become an interesting therapeutical target (8). Also, S100P, an EF-hand calcium-binding protein, was found to be another mediator of breast cancer malignancy and metastasis (9). There is also a difference in glycosylation in ER-positive and ER-negative tumors. Genes, involved in the synthesis of sialyl-Lewis x [sLe(x)] (FUT3, FUT4, ST3GAL6) are highly expressed in ER-negative tumors. ER-positive tumors with high expression of sLe(x) are correlated with bone metastasis, so that, for using sLe(x) as a tumor marker, the genetical surrounding has to be regarded (10). In a mouse model, it could be shown that S100A7/psoriasin is an important factor for breast tumorigenesis and metastasis by activation of proinflammatory pathways (11). Another association was found between diabetes mellitus and breast cancer, as glycosylation and oxidation of HDL, which are found in diabetic patients, lead to abnormal cell adhesion properties, in turn provoking metastasis formation, especially in the lungs and in the liver (12). In a further study, it was found that the actin-binding protein cofilin, which is O-GlcNAcetylated, is localized at the leading edge of lamellipodia, thereby playing an important role in cell migration. A loss of glycosylation of cofilin destabilizes the lamellipodia, impairing, or at least reducing metastatic potential of cells (13). That gives hints toward the role post-translational glycosylation is playing in tumor formation. Another rather recent finding in the field of glycosylation came from phyllodes tumors of the breast, an aggressively growing type of breast cancer: the gene expression of Axl and ST6GalNAcII was increased in malignant tumors, whereas the expression of Axl seems to be dependent on the expression of ST6GalNAcII. Their expression was correlated to tumor grading and the development of remote metastasis (14). And last but not least, also functional CD44 might be involved in breast cancer metastasis, representing a ligand for E-selectin, pointing again toward a role for CD44 in cell adhesion and motility (15).

## Molecules Associated with Glycosylation in Breast Cancer

### Helix Pomatia Lectin

Helix pomatia lectin recognizes mostly alpha-*N*-acetylgalactosamine residues, which are conferred to cells by an abnormal expression and activity of glycosyltransferases. The changes in glycosylation of cellular structures lead to an increased invasive capacity of these cells, thereby opening roads for the formation of remote metastasis, especially in local lymph nodes. HPA binding can therefore be regarded as a prognostic marker for clinical decisions like treatment strategies (16). Recently, the binding partners of HPA were analyzed more thoroughly. As major binding partners IgA1, complement factor C3, von Willebrand factor, alpha-2-macroglobulin, and IgM were identified. Hence, in the use of HPA in tumor characterization, blood group antigens have to be taken in consideration, or more in general, the genetical context of the patient has to be regarded, before deciding on therapeutical means (17). It was verified again by some glycotope expression analysis carried out in 2011 that HPA binding is a marker for metastasis (18). Furthermore, proteins that were identified as binding partners of HPA even give hints toward future outcome, as, for example, Integrin alpha 6 was the most abundant HPA glycoprotein in breast cancer cells with high metastatic potential (19).

### Mucin-1

Mucin-1, a large transmembrane protein, located on the apical surface of human epithelial cells, has already early been recognized as a carrier of aberrant O-glycosylation in tumor cells. The changes in glycosylation are accompanied by exposure of the protein core, which in term mediates the attachment of those cells to tissue at different sites, which is an important step in the formation of remote metastasis (20). This underglycosylation of MUC1 is also associated with a higher grading and might serve as a diagnostic marker (21). One protein, CIN85, was identified recently, which associates with MUC1 in tumor cells. The complex formed of these two proteins is a key mediator for cell migration and invasion, thereby promoting metastasis formation (22). Furthermore, it was found out that MUC1, MUC1-core, and TF are especially expressed in mucinous tumors (as well in primary tumors as in the corresponding metastases), while CA19-9, CA50, and CA242 seemed to be more characteristic for gastrointestinal tumors (23).

### Galectins

Galectins are endogenous galactoside-binding lectins with conserved features in the binding site. They are involved in cell adhesion and growth regulation. An increased binding of galectin-1 and reduced expression of galectin-3 or a reduced binding of both seemed to correlate with lymph node metastasis (24). Another correlation was found for galectin-3 and gelatinase B [matrix-metalloproteinase-9 (MMP-9)]. Cancer-associated isoforms of MMP-9 bound galectin-3 in a rather reduced manner than natural MMP-9 did. The role of galectin-3 is to bind laminin and MMP-9, thereby locating MMP-9 to the ECM. If this binding is reduced in tumor cells and MMP-9 is not bound to ECM, it cleaves ECM-proteins influencing cell adhesion properties in respect to metastasis formation (25). Another ligand for galectin-3 is cancer-associated MUC1. This binding is mediated by the Thomsen–Friedenreich antigen (TF-Ag), and it promotes cancer cell adhesion to the endothelium, contributing in this way to metastasis formation (26, 27).

## Influence of Gene Expression on Glycosylation and Metastasis

There are, of course, some more genes and their gene products, which play a role in metastasis formation in the context of glycosylation. In 1999, the adhesion-regulating molecule-1 (ARM-1) was identified by expression library screening from a murine lymphoma cell line. This gene codes for a type I transmembrane protein with O- and N-glycosylation sites, and shows increased expression in metastatic breast cancer cell lines in comparison to non-metastatic cells (28). Another gene/gene product involved in breast cancer metastasis seems to be extracellular matrix metalloproteinase inducer (EMMPRIN), which stimulates peritumoral fibroblasts to produce MMPs, which in turn contribute to tumor invasion. EMMPRIN isoforms are differentially glycosylated with Lewis x structures and could serve as therapeutic and diagnostic target by predicting negative side effects (29). A completely different mechanism is seen for Ribophorin II (RPN2). It is part of an *N*-oligosaccharide transferase complex, which is responsible for drug resistance in breast cancer cells. A lower expression of RPN2 results in a reduced glycosylation and delocalization of CD63. The localization of the multidrug resistance protein (MDR1) is regulated by CD63, which in turn plays a role in cancer malignancy by drug resistance and cell invasion (30).

## Glycosylation and Brain Metastasis

Galectin-3 is also a famous molecule in tumor progression. Its expression is negative in normal breast tissue, but is increased in breast cancer and brain metastases (31). Nevertheless, for the formation of metastases in the brain a rather sophisticated cascade of molecules is necessary to pass the blood–brain barrier. A specific mediator to overcome this barrier seems to be ST6GALNAC5, which is not only expressed in the brain but also in breast cancer cells, mediating the adhesion of cancer cells to brain endothelial cells by changing glycosylation patterns (32). Another glycosyltransferase, GALNT9, an initiator of O-glycosylation, also seems to play a role in breast-to-brain metastatic process, and may become a useful prognostic marker (33).

## Glycosylation in Cancer Control

### Breast Cancer Diagnosis

Alterations in glycosylation structures could serve as important diagnostic markers. In 2005, the role of beta-1,6-branched oligosaccharides, which are transferred to *N*-glycans by beta-1,6-*N*-acteylglucosaminyltransferase V, was shown by Handerson et al. The appearance of those oligosaccharide structures could be related to nodal metastasis, and are a predictor of poor outcome, independent of tumor size, grading, or patient age at primary diagnosis (34). Another marker for a poor clinical outcome is the occurrence of disseminated tumor cells (DTCs) in the bone marrow of breast cancer patients. DTCs also have aberrant glycosylation motifs, and can be detected by the presence of UDP-*N*-acetyl-d-galactosamine:polypeptide N-acetylgalactosaminyltransferase 6 (ppGalNAc-T6) mRNA in bone marrow aspirates by RT-PCR (35). Also, the receptor of advanced glycosylation end products (RAGE) and its soluble form (sRAGE) could be used in cancer diagnosis: patients with breast cancer at high stages show lower sRAGE levels, and these low levels might contribute to disease progression (36). After a digestion of the glycan pool of a cell with a combination of sialidase and beta-galactosidase, a digestion product was obtained in the form of a monogalactosylated triantennary structure containing alpha 1,3-linked fucose. This product was found twice as much in breast cancer patients than in samples of a healthy control group and even seems to be better biomarker than CA15-3 and CEA in predicting disease progression and occurrence of remote metastasis (37). Another marker predicting disease progression and a tendency toward lymph node metastasis is glycodelin, an endometrially secreted glycoprotein, which exists in different glycosylation states (38). In a mouse model, the changes in N-linked glycans during cancer progression were measured by matrix-assisted laser desorption/ionization Fourier transform-ion cyclotron mass spectrometry. The results were transferred to human serum samples, and an alteration in low-abundance high-mannose glycans could be seen in breast cancer patients, especially an elevation of a high-mannose type glycan was seen in mouse and human breast cancer sera, suggesting an incompletion of the glycosylation process (39). Another method for the analysis of glycosylation might be the detection of antibodies produced by the immune system against even minor changes in glycosylation structures. The advantage of this method is the earlier detection of antibodies in comparison to the antigen. Such a study was performed by Blixt et al. to analyze changes in Mucin-1 O-glycosylation from serum of breast cancer patients. Antibody levels were significantly higher in sera of cancer patients than in healthy control samples. High levels of core3MUC1 could even be correlated to a later onset of metastasis, thereby representing a promising new tumor marker (40). Another technique for the analysis of glycosylation is the conduction of lectin microarrays and a lectin-binding signature for breast cancer metastasis had already been described (41). Also, ELISAs using HPA could serve in cancer diagnostics. In a rather recent assay cadherin-5 glycosylation was thereby found to be a marker for breast cancer recurrence with a specificity of 90% (42). Mircoarray analysis of glycosylation-related genes in breast cancer samples lead to an interesting finding: in luminal cancers N-glycosylation seemed to be more prominent, while in triple-negative cancers O-glycosylation plays a more important role in tumor progression, pointing toward a different effect of glycosylation patterns (43). In further analyses using lectin histochemistry and tissue microarrays, it was shown that lectin binding correlates with parameters involved in tumor metastasis and significantly shorter overall survival (OAS), so that it could be concluded once more that glycosylation and the enzymes involved in glycosylation processes play an important role in cancer progression (44). Another example for those findings is the enzyme fucosyltransferase IV (FUT IV), which is associated with proliferation and metastasis of breast cancer and could serve as a new biomarker in the diagnosis and prognosis of breast cancer (45).

### Glycosylation in Breast Cancer Therapy

Further research was done in order to use glycosylation of cellular structures not only in cancer diagnosis and prognosis, but also for therapeutical means (46). As early as 2001, a development of anti-cancer vaccines on the basis of carbohydrate structures was attempted. Some glycosyl structures are already known to suppress metastasis and invasive potential, in some cases also the mechanisms for suppression are known and some anti-cancer vaccines were already reported. The great challenge in this field is to develop vaccines that provide a similar effect to that of tumor-associated carbohydrate antigens in respect to suppression of tumor progression (47). Such a target for cancer therapy could be the TF-Ag, which is expressed exclusively on the surface of tumor cells and has influence on cell adhesion and thereby on metastasis formation. Treatment of a mouse breast cancer model with JAA-F11, a monoclonal antibody to TF-Ag already led to reduced lung metastasis formation and improved prognosis. As natural antibodies against TF are produced from the body, the risk within such a vaccination for cancer patient is relatively small, so that it could be a rather promising therapeutic target (48). In line with TF, also antibodies against Tn seemed to have therapeutical meanings: a monoclonal antibody to Tn (2154F12A4) selectively recognized Tn and inhibited the adhesion of cancer cells to the lymphatic endothelium, and could thus inhibit lymphatic metastases (49). Another interesting fact was reported from Yuan et al. (50): they could show that a defucosylation impaired rolling of human breast cancer cells on a cell layer and increased simultaneously the flow speed of the tumor cells, so that it was concluded that reduced fucosylation levels impaired interaction of tumor cells with endothelial cells, modulating tumor progression and thus being also a target for tumor therapy (50).

## Conclusion

Glycosylation plays an important role in a number of processes leading to breast cancer metastasis. A lot of research was already done on this topic in the last 25 years, clarifying the role of some glycosyltransferases, examining the function of altered glycosylation for cell adhesion and motility properties, ending up in the analysis of different glycosylation patterns for metastasis formation. But still lots of work needs to be done to understand better the mechanisms of metastasis formation and the involvement of glycosylation within, to profit from this knowledge in terms of cancer diagnosis and treatment options. Further research in this field could open new roads in breast cancer diagnosis as certain glycosylation pattern could possibly be related to clinical outcome and could help to analyze, which changes happened in the tumor cells. Emanating from this knowledge also cancer therapeutics could profit from the analysis of glycosylation patterns. The examination of glycosylation patterns could lead to a more personalized tumor therapy, in which side effects could be reduced while the efficiency of a therapy is increased. Therefore, glycosylation might play an important role in cancer combating in the future.

## Conflict of Interest Statement

The authors declare that the research was conducted in the absence of any commercial or financial relationships that could be construed as a potential conflict of interest.

## Abbreviations

Ag, antigen; ARM-1, adhesion-regulating molecule; CA15-3, cancer antigen; CD44, cluster of differentiation; CEA, carcinoembryonic antigen; CTCs, circulating tumor cells; DSA, *Datura stramonium* agglutinin; DTCs, disseminated tumor cells; ECM, extracellular matrix; ELISA, enzyme-linked immunosorbent assay; EMMPRIN, extracellular metalloproteinase inhibitor; EMT, epithelial to mesenchymal transition; ER, estrogen receptor; FUT, fucosyltransferase; GCNT, glucosaminyl (*N*-acetyl) transferase; HDL, high density lipoprotein; HPA, helix pomatia agglutinin; MDR, multidrug resistance protein; MMP, matrix-metalloproteinase; RAGE, receptor of advanced glycosylation end products; RPN, ribophorin; T, Thomsen–Friedenreich antigen; Tn, Thomsen-nouvelle antigen; TGF, transforming growth factor.

## References

[1] Brooks SA Carter TM. N-acetylgalactosamine, N-acetylglucosamine and sialic acid expression in primary breast cancers. Acta Histochem (2001) 103:37–51. 10.1078/0065-1281-00576 11252626

[2] Brooks SA Leathem AJ. Prediction of lymph node involvement in breast cancer by detection of altered glycosylation in the primary tumour. Lancet (1991) 338:71–4. 10.1016/0140-6736(91)90071-V 1712062

[3] Hiraizumi S Takasaki S Ohuchi N Harada Y Nose M Mori S Altered glycosylation of membrane glycoproteins associated with human mammary carcinoma. Jpn J Cancer Res (1992) 83:1063–72. 10.1111/j.1349-7006.1992.tb02723.x 1452459 PMC5918672

[4] Springer GF. Immunoreactive T and Tn epitopes in cancer diagnosis, prognosis, and immunotherapy. J Mol Med (1997) 75:594–602. 10.1007/s001090050144 9297627

[5] Duffy MJ Shering S Sherry F McDermott E O’Higgins N. CA 15-3: a prognostic marker in breast cancer. Int J Biol Markers (2000) 15:330–3. 11192829 10.1177/172460080001500410

[6] Lin S Kemmner W Grigull S Schlag PM. Cell surface alpha 2,6 sialylation affects adhesion of breast carcinoma cells. Exp Cell Res (2002) 276:101–10. 10.1006/excr.2002.5521 11978012

[7] Saldova R Reuben JM Abd Hamid UM Rudd PM Cristofanilli M. Levels of specific serum N-glycans identify breast cancer patients with higher circulating tumor cell counts. Ann Oncol (2011) 22:1113–9. 10.1093/annonc/mdq570 21127012

[8] Zhang H Meng F Wu S Kreike B Sethi S Chen W Engagement of I-branching {beta}-1, 6-N-acetylglucosaminyltransferase 2 in breast cancer metastasis and TGF-{beta} signaling. Cancer Res (2011) 71:4846–56. 10.1158/0008-5472.CAN-11-0414 21750175 PMC3903410

[9] Gibadulinova A Tothova V Pastorek J Pastorekova S. Transcriptional regulation and functional implication of S100P in cancer. Amino Acids (2011) 41:885–92. 10.1007/s00726-010-0495-5 20155429

[10] Julien S Ivetic A Grigoriadis A QiZe D Burford B Sproviero D Selectin ligand sialyl-Lewis x antigen drives metastasis of hormone-dependent breast cancers. Cancer Res (2011) 71:7683–93. 10.1158/0008-5472.CAN-11-1139 22025563 PMC6485480

[11] Nasser MW Qamri Z Deol YS Ravi J Powell CA Trikha P S100A7 enhances mammary tumorigenesis through upregulation of inflammatory pathways. Cancer Res (2012) 72:604–15. 10.1158/0008-5472.CAN-11-0669 22158945 PMC3271140

[12] Pan B Ren H He Y Lv X Ma Y Li J HDL of patients with type 2 diabetes mellitus elevates the capability of promoting breast cancer metastasis. Clin Cancer Res (2012) 18:1246–56. 10.1158/1078-0432.CCR-11-0817 22261802

[13] Huang X Pan Q Sun D Chen W Shen A Huang M O-GlcNAcylation of cofilin promotes breast cancer cell invasion. J Biol Chem (2013) 288:36418–25. 10.1074/jbc.M113.495713 24214978 PMC3868755

[14] Ren D Li Y Gong Y Xu J Miao X Li X Phyllodes tumor of the breast: role of Axl and ST6GalNAcII in the development of mammary phyllodes tumors. Tumour Biol (2014) 35:9603–12. 10.1007/s13277-014-2254-9 24961352

[15] Shirure VS Liu T Delgadillo LF Cuckler CM Tees DF Benencia F CD44 variant isoforms expressed by breast cancer cells are functional E-selectin ligands under flow conditions. Am J Physiol Cell Physiol (2015) 308:C68–78. 10.1152/ajpcell.00094.2014 25339657 PMC4281670

[16] Brooks SA. The involvement of helix pomatia lectin (HPA) binding N-acetylgalactosamine glycans in cancer progression. Histol Histopathol (2000) 15:143–58. 10.1021/bi061254l 10668205

[17] Welinder C Jansson B Ferno M Olson H Baldetorp B. Expression of helix pomatia lectin binding glycoproteins in women with breast cancer in relationship to their blood group phenotypes. J Proteome Res (2009) 8:782–7. 10.1021/pr800444b 18998722

[18] Schnegelsberg B Schumacher U Valentiner U. Lectin histochemistry of metastasizing and non-metastasizing breast and colon cancer cells. Anticancer Res (2011) 31:1589–97. 10.1039/C3MB70359B 21617214

[19] Rambaruth ND Greenwell P Dwek MV. The lectin helix pomatia agglutinin recognizes O-GlcNAc containing glycoproteins in human breast cancer. Glycobiology (2012) 22:839–48. 10.1093/glycob/cws051 22322011

[20] Ciborowski P Finn OJ. Non-glycosylated tandem repeats of MUC1 facilitate attachment of breast tumor cells to normal human lung tissue and immobilized extracellular matrix proteins (ECM) in vitro: potential role in metastasis. Clin Exp Metastasis (2002) 19:339–45. 10.1023/A:1015590515957 12090474

[21] Ghosh SK Pantazopoulos P Medarova Z Moore A. Expression of underglycosylated MUC1 antigen in cancerous and adjacent normal breast tissues. Clin Breast Cancer (2013) 13:109–18. 10.1016/j.clbc.2012.09.016 23122537 PMC3578066

[22] Cascio S Farkas AM Hughey RP Finn OJ. Altered glycosylation of MUC1 influences its association with CIN85: the role of this novel complex in cancer cell invasion and migration. Oncotarget (2013) 4:1686–97. 10.18632/oncotarget.1265 24072600 PMC3858555

[23] Gunkel L Mylonas I Richter DU Makovitzky J. Immunohistochemical studies of mucinous mammary carcinomas and their metastases. Anticancer Res (2005) 25:1755–9. 16033095

[24] Andre S Kojima S Yamazaki N Fink C Kaltner H Kayser K Galectins-1 and -3 and their ligands in tumor biology. Non-uniform properties in cell-surface presentation and modulation of adhesion to matrix glycoproteins for various tumor cell lines, in biodistribution of free and liposome-bound galectins and in their expression by breast and colorectal carcinomas with/without metastatic propensity. J Cancer Res Clin Oncol (1999) 125:461–74. 10480338 10.1007/s004320050303 PMC12172401

[25] Fry SA Van den Steen PE Royle L Wormald MR Leathem AJ Opdenakker G Cancer-associated glycoforms of gelatinase B exhibit a decreased level of binding to galectin-3. Biochemistry (2006) 45:15249–58. 10.1021/bi061254l 17176047

[26] Yu LG Andrews N Zhao Q McKean D Williams JF Connor LJ Galectin-3 interaction with Thomsen-Friedenreich disaccharide on cancer-associated MUC1 causes increased cancer cell endothelial adhesion. J Biol Chem (2007) 282:773–81. 10.1074/jbc.M606862200 17090543

[27] Simone G Malara N Trunzo V Renne M Perozziello G Di Fabrizio E Galectin-3 coats the membrane of breast cells and makes a signature of tumours. Mol Biosyst (2014) 10:258–65. 10.1039/c3mb70359b 24281352

[28] Simins AB Weighardt H Weidner KM Weidle UH Holzmann B. Functional cloning of ARM-1, an adhesion-regulating molecule upregulated in metastatic tumor cells. Clin Exp Metastasis (1999) 17:641–8. 10.1023/A:1006790912877 10919708

[29] Riethdorf S Reimers N Assmann V Kornfeld JW Terracciano L Sauter G High incidence of EMMPRIN expression in human tumors. Int J Cancer (2006) 119:1800–10. 10.1002/ijc.22062 16721788

[30] Tominaga N Hagiwara K Kosaka N Honma K Nakagama H Ochiya T. RPN2-mediated glycosylation of tetraspanin CD63 regulates breast cancer cell malignancy. Mol Cancer (2014) 13:134. 10.1186/1476-4598-13-134 24884960 PMC4070641

[31] Mayoral MA Mayoral C Meneses A Villalvazo L Guzman A Espinosa B Identification of galectin-3 and mucin-type O-glycans in breast cancer and its metastasis to brain. Cancer Invest (2008) 26:615–23. 10.1080/07357900701837051 18584353

[32] Bos PD Zhang XH Nadal C Shu W Gomis RR Nguyen DX Genes that mediate breast cancer metastasis to the brain. Nature (2009) 459:1005–9. 10.1038/nature08021 19421193 PMC2698953

[33] Pangeni RP Channathodiyil P Huen DS Eagles LW Johal BK Pasha D The GALNT9, BNC1 and CCDC8 genes are frequently epigenetically dysregulated in breast tumours that metastasise to the brain. Clin Epigenetics (2015) 7:57. 10.1186/s13148-015-0089-x 26052355 PMC4457099

[34] Handerson T Camp R Harigopal M Rimm D Pawelek J. Beta1,6-branched oligosaccharides are increased in lymph node metastases and predict poor outcome in breast carcinoma. Clin Cancer Res (2005) 11:2969–73. 10.1158/1078-0432.CCR-04-2211 15837749

[35] Freire T Berois N Sonora C Varangot M Barrios E Osinaga E. UDP-N-acetyl-D-galactosamine:polypeptide N-acetylgalactosaminyltransferase 6 (ppGalNAc-T6) mRNA as a potential new marker for detection of bone marrow-disseminated breast cancer cells. Int J Cancer (2006) 119:1383–8. 10.1002/ijc.21959 16596643

[36] Tesarova P Kalousova M Jachymova M Mestek O Petruzelka L Zima T. Receptor for advanced glycation end products (RAGE) – soluble form (sRAGE) and gene polymorphisms in patients with breast cancer. Cancer Invest (2007) 25:720–5. 10.1080/07357900701560521 18058469

[37] Abd Hamid UM Royle L Saldova R Radcliffe CM Harvey DJ Storr SJ A strategy to reveal potential glycan markers from serum glycoproteins associated with breast cancer progression. Glycobiology (2008) 18:1105–18. 10.1093/glycob/cwn095 18818422

[38] Scholz C Toth B Barthell E Mylonas I Weissenbacher T Friese K Glycodelin expression in correlation to grading, nodal involvement and steroid receptor expression in human breast cancer patients. Anticancer Res (2010) 30:1599–603. 20592348

[39] de Leoz ML Young LJ An HJ Kronewitter SR Kim J Miyamoto S High-mannose glycans are elevated during breast cancer progression. Mol Cell Proteomics (2011) 10:M110002717. 10.1074/mcp.M110.002717 21097542 PMC3013453

[40] Blixt O Bueti D Burford B Allen D Julien S Hollingsworth M Autoantibodies to aberrantly glycosylated MUC1 in early stage breast cancer are associated with a better prognosis. Breast Cancer Res (2011) 13:R25. 10.1186/bcr2841 21385452 PMC3219186

[41] Fry S Afrough B Leathem A Dwek M. Lectin array-based strategies for identifying metastasis-associated changes in glycosylation. Methods Mol Biol (2012) 878:267–72. 10.1007/978-1-61779-854-2_18 22674140

[42] Fry SA Sinclair J Timms JF Leathem AJ Dwek MV. A targeted glycoproteomic approach identifies cadherin-5 as a novel biomarker of metastatic breast cancer. Cancer Lett (2013) 328:335–44. 10.1016/j.canlet.2012.10.011 23079531

[43] Milde-Langosch K Karn T Schmidt M Zu Eulenburg C Oliveira-Ferrer L Wirtz RM Prognostic relevance of glycosylation-associated genes in breast cancer. Breast Cancer Res Treat (2014) 145:295–305. 10.1007/s10549-014-2949-z 24737166

[44] Milde-Langosch K Schutze D Oliveira-Ferrer L Wikman H Muller V Lebok P Relevance of betaGal-betaGalNAc-containing glycans and the enzymes involved in their synthesis for invasion and survival in breast cancer patients. Breast Cancer Res Treat (2015) 151:515–28. 10.1007/s10549-015-3425-0 25975956

[45] Yan X Lin Y Liu S Aziz F Yan Q. Fucosyltransferase IV (FUT4) as an effective biomarker for the diagnosis of breast cancer. Biomed Pharmacother (2015) 70:299–304. 10.1016/j.biopha.2014.12.048 25776515

[46] Vlad AM Finn OJ. Glycoprotein tumor antigens for immunotherapy of breast cancer. Breast Dis (2004) 20:73–9. 15687709 10.3233/bd-2004-20109

[47] Hakomori S. Tumor-associated carbohydrate antigens defining tumor malignancy: basis for development of anti-cancer vaccines. Adv Exp Med Biol (2001) 491:369–402. 10.1007/978-1-4615-1267-7_24 14533809

[48] Almogren A Abdullah J Ghapure K Ferguson K Glinsky VV Rittenhouse-Olson K. Anti-Thomsen-Friedenreich-Ag (anti-TF-Ag) potential for cancer therapy. Front Biosci (2012) 4:840–63. 10.2741/S304 22202095

[49] Danussi C Coslovi A Campa C Mucignat MT Spessotto P Uggeri F A newly generated functional antibody identifies Tn antigen as a novel determinant in the cancer cell-lymphatic endothelium interaction. Glycobiology (2009) 19:1056–67. 10.1093/glycob/cwp085 19528665 PMC2736043

[50] Yuan K Kucik D Singh RK Listinsky CM Listinsky JJ Siegal GP. Alterations in human breast cancer adhesion-motility in response to changes in cell surface glycoproteins displaying alpha-L-fucose moieties. Int J Oncol (2008) 32:797–807. 18360707 PMC2671470

